# Diminished Neural Processing of Aversive and Rewarding Stimuli During Selective Serotonin Reuptake Inhibitor Treatment

**DOI:** 10.1016/j.biopsych.2009.11.001

**Published:** 2010-03-01

**Authors:** Ciara McCabe, Zevic Mishor, Philip J. Cowen, Catherine J. Harmer

**Affiliations:** Department of Psychiatry, Warneford Hospital, University of Oxford, Oxford, United Kingdom

**Keywords:** Antidepressants, depression, fMRI, reward, SSRI, ventral striatum

## Abstract

**Background:**

Selective serotonin reuptake inhibitors (SSRIs) are popular medications for anxiety and depression, but their effectiveness, particularly in patients with prominent symptoms of loss of motivation and pleasure, has been questioned. There are few studies of the effect of SSRIs on neural reward mechanisms in humans.

**Methods:**

We studied 45 healthy participants who were randomly allocated to receive the SSRI citalopram, the noradrenaline reuptake inhibitor reboxetine, or placebo for 7 days in a double-blind, parallel group design. We used functional magnetic resonance imaging to measure the neural response to rewarding (sight and/or flavor of chocolate) and aversive stimuli (sight of moldy strawberries and/or an unpleasant strawberry taste) on the final day of drug treatment.

**Results:**

Citalopram reduced activation to the chocolate stimuli in the ventral striatum and the ventral medial/orbitofrontal cortex. In contrast, reboxetine did not suppress ventral striatal activity and in fact increased neural responses within medial orbitofrontal cortex to reward. Citalopram also decreased neural responses to the aversive stimuli conditions in key “punishment” areas such as the lateral orbitofrontal cortex. Reboxetine produced a similar, although weaker effect.

**Conclusions:**

Our findings are the first to show that treatment with SSRIs can diminish the neural processing of both rewarding and aversive stimuli. The ability of SSRIs to decrease neural responses to reward might underlie the questioned efficacy of SSRIs in depressive conditions characterized by decreased motivation and anhedonia and could also account for the experience of emotional blunting described by some patients during SSRI treatment.

Selective serotonin reuptake inhibitors (SSRIs) have become the mainstay of pharmacologic treatment for depressive and anxiety disorders and hold a preeminent position in the global antidepressant market (1). SSRIs have achieved this predominance through their reasonable tolerance and broad range of clinical indications; however, concerns about their efficacy and behavioral toxicity persist (2,3). For example, although SSRIs appear effective at attenuating pervasive negative affect, a key clinical feature of anxiety and depressive disorders (4), their ability to improve diminished positive affect by relieving symptoms of low energy, decreased motivation, and anhedonia has been questioned (5). Related to this are reports that some patients associate SSRI treatment with an experience of emotional blunting through which emotional responses to both aversive and pleasurable experiences are diminished (6,7). Experimental studies in animals and humans indicate that serotonin pathways exert an inhibitory influence over neural systems mediating both positive and negative affective processes (8). Thus, increases in serotonin function produced by SSRIs could produce a form of “emotional constraint” in which the salience of both rewarding and aversive stimuli is lost (8). In view of the widespread use of SSRIs, such an effect could have considerable personal and social implications.

Investigating the effect of SSRIs on emotional responses in depressed patients is difficult because loss of pleasure (anhedonia) may persist even during clinical remission (9). In addition, modest degrees of emotional blunting might be difficult for individuals to detect or report subjectively. In this study, we used functional magnetic resonance imaging (fMRI) to measure the neural basis of primary rewarding and aversive stimuli in healthy volunteers who received short-term treatment with the widely used SSRI citalopram. As a comparison, we studied the effects of a similar course of treatment with reboxetine, an antidepressant drug that selectively inhibits reuptake of the catecholamine neurotransmitter noradrenaline. Clinically, catecholamine-potentiating agents such as reboxetine have been suggested by some to be more effective at treating symptoms such as anhedonia in depression (5,10) and produce more robust effects in reinforcement paradigms in animal experimental studies than SSRIs (11). We therefore predicted that citalopram treatment would lower the neural response to both rewarding and aversive stimuli in the key reward circuitry of ventral striatum, medial prefrontal, and orbitofrontal cortices in which we and others have shown to be sensitive to unconditioned rewarding and aversive stimuli (12,13) whereas we expected that neural responses to rewarding stimuli would be preserved following reboxetine treatment.

## Methods and Materials

### Participants

Forty-five healthy volunteers were randomized to receive 7 days oral treatment with citalopram (20 mg/day, *n* = 15), reboxetine (4 mg twice daily, *n* = 15), or placebo (*n* = 15) in a double-blind between-groups design. Medication was taken twice a day, once in the morning and once in the evening, to maintain blinding. Ethical approval was provided by the Oxford Research Ethics Committee B, and written informed consent was obtained from all participants before screening and after a complete description of the study was given. Exclusion criteria for all subjects were current or past Axis 1 disorder on the Structured Clinical Interview for DSM-IV (14) and any contraindications to MRI (e.g., pacemaker, mechanical heart valve, hip replacement, metal implants).

None of the participants took current medication apart from the contraceptive pill. Before drug administration and to ensure group matching, baseline information was collected using the Beck Depression Inventory (15), State-Trait Anxiety Inventory (16), the Fawcett-Clarke Pleasure Scale (17), and the Snaith-Hamilton Pleasure Scale (18). The participants also completed a “chocolate questionnaire” to measure liking, craving, and frequency of eating chocolate (19) and the Eating Attitudes Test questionnaire, which measures attitudes to food (20). Body mass index (BMI) was also calculated for each volunteer. To assess the effects of the treatment, the following questionnaires were taken before and after the treatment; visual analogue scales of happiness, sadness, anger, disgust, alertness, and anxiety, and the State-Trait Anxiety Inventory (16) (Table 1).

### Overall Design

We compared brain responses to reward -related and aversive stimuli across the three drug groups. Each of the following conditions were applied nine times in a randomized order (Table S1 in Supplement 1): chocolate in the mouth, chocolate picture, chocolate in the mouth with chocolate picture, strawberry in the mouth, strawberry picture, strawberry in the mouth with strawberry picture. Subjective effects of the stimuli were measured by psychophysical ratings of “pleasantness,” “intensity,” and “wanting” made on every trial by the subjects during the fMRI acquisition. The participants were instructed not to eat chocolate for 24 hours before the scan and to eat only a small lunch on the day of scanning. Mood state was recorded on the study day with the Beck Depression Inventory (15).

### Stimuli

Stimuli were delivered to the subject's mouth through three Teflon tubes (one for the tasteless rinse control described later, one for chocolate taste, and one for strawberry taste); the tubes were held between the lips. Each tube was connected to a separate reservoir through a syringe and a one-way syringe activated check valve (Model 14044-5, World Precision Instruments, Stevenage, United Kingdom), which allowed .5 mL of any stimulus to be delivered manually at the time indicated by the computer. The chocolate was formulated to be liquid at room temperature, with a list of the six stimulus conditions described in Table S1 in Supplement 1. A control tasteless solution .5 mL of a saliva-like rinse solution (25 × 10^−3^ mol/L KCl and 2.5 × 10^−3^ mol/L NaHCO_3_ in distilled H_2_O) was used between trials (Table S1 in Supplement 1), which when subtracted from the effects of the other stimuli allowed somatosensory and any mouth movement effects to be subtracted from the effects produced by the other oral stimuli (21,22). This allowed the taste, texture, and olfactory areas to be shown independently of any somatosensory effects produced by introducing a fluid into the mouth (21–24). The aversive stimulus was a strawberry drink (Rosemount Pharmaceuticals, Leeds, United Kingdom), which was rated as intense as the chocolate but unpleasant in valence (13). Both the liquid chocolate and the strawberry had approximately the same sweetness and texture, which enabled them to pass freely through the Teflon delivery tubes.

### Experimental Procedure

At the beginning of each trial, one of the six stimuli chosen by random permutation was presented. If the trial involved an oral stimulus, this was delivered in a .5-mL aliquot to the subject's mouth. At the same time, at the start of the trial, a visual stimulus was presented, which was the picture of chocolate, of moldy strawberries, or a gray control image of approximately the same intensity. The image was turned off after 7 sec, at which time a small green cross appeared on a visual display to indicate to the subject to swallow what was in the mouth. After a delay of 2 sec, the subject was asked to rate each of the stimuli for “pleasantness” on that trial (with + 2 being very pleasant and −2 very unpleasant), for “intensity” on that trial (0 to +4), and for “wanting” (+ 2 for wanting very much, 0 for neutral, and −2 for very much not wanting). The ratings were made with a visual analog scale in which the subject moved the bar to the appropriate point on the scale using a button box. After the last rating, the gray visual stimulus indicated the delivery of the tasteless control solution, which was also used as a rinse between stimuli; this was administered in exactly the same way as a test stimulus, and after 7 sec the subject was cued by the green cross to swallow. The tasteless control was always accompanied by the gray visual stimulus. On trials in which only the picture of chocolate was shown, there was no rinse, but the gray visual stimulus was shown to allow an appropriate contrast as described subsequently. There was then a 2-sec delay period that allowed for swallowing followed by a 1-sec gap until the start of the next trial. A trial was repeated for each of the six stimulus conditions shown in Table S1 in Supplement 1, and the whole cycle was repeated nine times. The instruction given to the subject was (on oral delivery trials) to move the tongue once as soon as a stimulus or tasteless solution was delivered to distribute the solution around the mouth to activate the receptors for taste and smell and then to keep still for the remainder of the 7-sec period until the green cross was shown, when the subject could swallow. This procedure has been shown to allow taste effects to be demonstrated clearly with fMRI, using the procedure of subtracting any activations produced by the tasteless control from those produced by a taste or other stimulus (21–24).

### fMRI Scan

The experimental protocol consisted of an event-related interleaved design using in random permuted sequence the six stimuli as described above and shown in Table S1 in Supplement 1. Images were acquired with a 3.0 T Varian/Siemens whole-body scanner at the Centre for Functional Magnetic Resonance Imaging at Oxford, where T2*-weighted echo planar imaging (EPI) slices were acquired every 2 sec (repetition time = 2). Imaging parameters were selected to minimize susceptibility and distortion artifact in the orbitofrontal cortex (25). Coronal slices (25) with in-plane resolution of 3 × 3 mm and between plane spacing of 4 mm were obtained. The matrix size was 64 × 64, and the field of view was 192 × 192 mm. Acquisition was carried out during the task performance, yielding 972 volumes in total. A whole brain T2*-weighted EPI volume of these dimensions and an anatomic T1 volume with coronal plane slice thickness 3 mm and in-plane resolution of 1.0 × 1.0 mm was also acquired.

### fMRI Analysis

The imaging data were analyzed using SPM5 (http://www.fil.ion.ucl.ac.uk/spm/). Preprocessing of the data used SPM5 realignment, reslicing with sinc interpolation, normalization to the Montreal Neurological Institute coordinate system, and spatial smoothing with a 6-mm full-width-at-half-maximum isotropic Gaussian kernel and global scaling (26). The time series at each voxel were low-pass filtered with a hemodynamic response kernel. Time series nonsphericity at each voxel was estimated and corrected for (27), and a high-pass filter with a cut-off period of 128 sec was applied. In the single-event design, a general linear model was then applied to the time course of activation in which stimulus onsets were modeled as single impulse response functions and then convolved with the canonical hemodynamic response function (HRF) (28). Linear contrasts were defined to test specific effects. Time derivatives were included in the basis functions set. Following smoothness estimation (29), linear contrasts of parameter estimates were defined to test the specific effects of each condition with each individual dataset. Voxel values for each contrast resulted in a statistical parametric map of the corresponding *t* statistic, which was then transformed into the unit normal distribution (SPM *Z*). The statistical parametric maps from each individual data set were then entered into second-level, random-effects analyses accounting for both scan-to-scan and subject-to-subject variability. To assess the between-group differences for each condition, a repeated-measures factorial design was used with Group as the between-group factor (three levels: citalopram, reboxetine, and placebo) and condition as the within-subject factor (six levels) (30). This was followed up with one-sample *t* tests to examine simple main effects of group.

SPM converts the *t* statistics to *Z* scores. For the between-group analyses, we report *p* values for each cluster within regions of a priori hypotheses (see Introduction), thresholded at *p* = .001 and fully corrected for the number of comparisons (resels) in the entire brain volume (“whole-brain” multiple comparisons for which *p* < .05 family-wise error. We report small volume corrections for brain regions in which we had an a priori hypothesis (see Introduction) as follows: pregenual cingulate cortex [−4 30 2], ventral striatum (−10 6 −2) and medial prefrontal cortex [0 54–12] (13,31). Peaks within 20 mm of these and that had a *p* value of at least *p* < .002 uncorrected in the whole brain analysis and with a cluster threshold of 30 contiguous voxels (*k* = 30) had applied small volume (false discovery rate) corrections for multiple comparisons (29) with a radius corresponding to the full width at half maximum of the spatial smoothing filter used. Plots of parameter estimates are extracted using the volume of interest eigen variates tool in SPM5 with a sphere of 6 mm around the peak voxel identified from the significant contrast of interest in the key areas described in our a priori hypotheses—namely, the ventral striatum and orbitofrontal cortex. For illustration purposes only, Wake Forest University Pick Atlas (32) was used to display activations (http://www.fmri.wfubmc.edu/cms/software). Coordinates of the activations are listed in the stereotactic space of the Montreal Neurological Institute's ICBM152 brain (Tables 2 and 3; Table S3 in Supplement 1).

## Results

### Demographic Details and Mood Ratings

There were no significant differences among the three groups as determined by one-way analyses of variance (ANOVAs) for age, sex, body mass index, chocolate liking, and attitudes toward food (Eating Attitudes Test), *p* > .07 (Table 1). There were no significant differences between the three groups as determined by one-way ANOVAs for measures of anhedonia (Snaith-Hamilton Pleasure Scale, Fawcett-Clarke Pleasure Scale) or mood (Beck Depression Inventory), *p* > .1 (Table 1). There were no significant differences between the three groups over the 7-day experimental period on visual analogue scales (alertness, disgust, drowsiness, anxiety, happiness, nausea, and sadness) as determined by repeated-measures ANOVAs, *p* > .1 (Table S2 in Supplement 1).

### Ratings of Stimuli

Ratings of pleasantness, intensity, and wanting for the stimuli were obtained during the scanning on each trial for every condition. All subjects rated the strawberry picture and taste as unpleasant and the chocolate stimuli as pleasant. Using repeated-measures ANOVA with Ratings as a first factor with three levels (pleasantness, intensity, and wanting) and Condition as a second factor with six levels (Table S1 in Supplement 1 for the six condition levels), there was no significant main effect of Group [*F*(1,42) = .149, *p* = .95] or Condition by Group interaction, [*F*(1,42) = .32, *p* = .96; Table S4 in Supplement 1].

### fMRI Responses

Table S3 in Supplement 1 provides a summary of the results for each contrast across all subjects to indicate the main effect of task. Tables 2 and 3 provide a summary of the results of the interaction with Group.

### Main Effect of Task

As expected, the taste stimuli of chocolate and strawberry activated an overlapping region of the anterior insula (i.e., the primary taste cortex) in all subjects (Table S3 in Supplement 1). The rewarding stimuli chocolate taste and chocolate picture activated reward-relevant circuitry, including the ventral striatum, the cingulate cortex, and the mid-orbitofrontal cortex. By contrast, the unpleasant stimuli of strawberry taste and sight of the moldy strawberries activated areas involved in aversive processing, including the lateral orbitofrontal cortex extending into the insula cortex and the caudate (Table S3 in Supplement 1).

### Chocolate Reward: Sight and Taste

**Figure 1:**
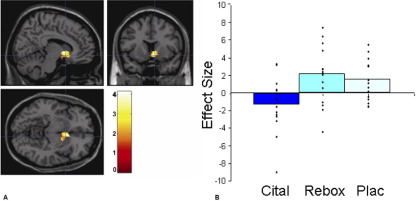
**(A)** Sight of chocolate condition (placebo [Plac] > citalopram [Cital]). Axial, sagittal, and coronal image of increased ventral striatal activation in the placebo group compared with the citalopram group ([6 14 −8], *z* = 3.79, *p* < .001, family-wise error corrected). Paramenter estimates from 6-mm sphere centered at 14 6 −8 for citalopram, reboxetine (Rebox), and placebo.

**Figure 2:**
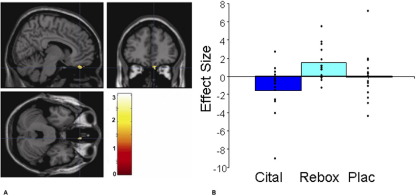
**(A)** Chocolate in the mouth with the sight of chocolate (reboxetine [Rebox] > placebo [Plac]): axial, sagittal, and coronal image of increased medial orbitofrontal cortex/frontal pole activation in the reboxetine group compared with the placebo ([10 42 −30], *z* = 3.01, *p* = .05 false discovery rate small volume corrected). **(B)** Parameter estimates from 6-mm sphere centered at 10 42 −30 for citalopram (Cital), reboxetine, and placebo.

The citalopram group, compared with placebo and reboxetine, showed less blood oxygen level–dependent (BOLD) activation to the sight and taste of chocolate in areas known to play a key role in reward, including the ventral striatum (Figure 1A and 1B) and the ventral medial orbitofrontal cortex. This activation was not in an area of signal dropout, confirmed by overlying the activation cluster on individual EPI data. There were no areas where the citalopram group showed greater BOLD activation relative to placebo or reboxetine for any of the conditions (Tables 2 and Table 3). Relative to placebo, the reboxetine group had enhanced BOLD activation to the chocolate in the medial orbitofrontal/frontal pole region (Figure 2A and 2B).

### Aversive Strawberry: Sight and Taste

**Figure 3:**
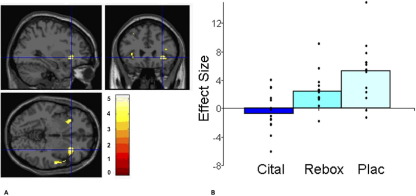
**(A)** Sight of moldy strawberries condition (placebo [Plac] > citalopram [Cital]): axial, sagittal, and coronal image of increased lateral orbitofrontal cortex activation in the placebo group compared with the citalopram group ([32 26 −4], *z* = 5.12, *p* = .002, family-wise error corrected). **(B)** Parameter estimates from 6-mm sphere centered at 32 26 −4 for citalopram, reboxetine (Rebox), and placebo.

**Figure 4:**
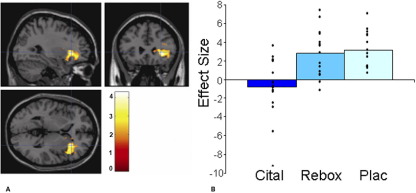
**(A)** Strawberry in the mouth with the sight of moldy strawberries (placebo [Plac] > citalopram [Cital]): axial, sagittal, and coronal image of increased lateral orbitofrontal cortex activation in the placebo group compared with the citalopram group ([30 26 2], *z* = 4.22, *p* < .001, family-wise error corrected). **(B)** Parameter estimates from 6-mm sphere centered at 30 26 2 for citalopram, reboxetine, and placebo.

The citalopram group relative to placebo showed less BOLD activation to the sight and taste of strawberry in areas known to play a key role in processing aversive stimuli, including the lateral orbitofrontal cortex and insula as illustrated in Figure 3A and 3B and Figure 4A and 4B. Relative to placebo, the reboxetine group also had less BOLD activation to the unpleasant strawberry taste in the lateral orbitofrontal cortex (Table 2).

## Discussion

Our findings are the first to show that 7-days treatment with an SSRI diminishes the neural processing of both rewarding and aversive stimuli. This is consistent with the proposal that SSRI treatment might produce a general constraint of emotional response (8) rather than simply decreasing the emotional impact of aversive stimuli. Such an effect could underlie the postulated modest antidepressant efficacy of SSRIs in patients whose depression is characterized by loss of motivation and pleasure (4) in whom abnormalities in neural reward mechanisms have been demonstrated in functional imaging studies (33,34). It has been suggested that such patients may benefit more from the use of catecholamine-potentiating antidepressants (4,10), which agrees with the effects of reboxetine seen here to increase some aspects of reward processing.

As expected, citalopram treatment had less BOLD activation to the aversive stimuli, consistent with the ability of SSRIs to diminish negative affect across a range of psychiatric disorders (4,10). Interestingly, reboxetine, seemed to have less effect on aversive responses than citalopram. This is in line with clinical observations that catecholamine-potentiating agents appear less effective than SSRIs at reducing distress and negative affect and have less overall utility, for example, in the management of anxiety disorders (5,35).

The ability of SSRIs to limit the neural activation to reward may therefore support the clinical reports that SSRI treatment is associated not only with experiences of diminished negative affect (less sadness, less ability to cry) (36) but in some patients also with diminished positive affect (decreased sexual pleasure and feelings of joy) (6,7). In SSRI-treated clinical populations who report diminished positive affect, it is difficult to disentangle possible effects of subclinical depressive symptomatology or trait disturbances in reward mechanisms from those of concomitant SSRI treatment. For example, we have shown that unmedicated, clinically remitted depressed patients demonstrate blunted striatal responses to chocolate reward (13). However, the present study of healthy subjects indicates that SSRI treatment can itself attenuate the neural processing of reward as well as that of aversive stimuli. It is conceivable, however, that in acutely depressed patients, SSRI treatment might produce a different effect on the neural processing of reward than that seen in healthy volunteers. Further studies are necessary to explore this possibility.

The time course of our study (7 days) was limited compared with the duration of clinical treatment with antidepressants, and it is therefore also relevant to study the effect of SSRIs on the neural basis of reward after longer treatment periods. However, a meta-analysis of clinical trials has shown that, relative to placebo, SSRIs significantly decrease depressive symptomatology after 1 week of treatment (37), and indeed the effect size of active treatment relative to placebo is numerically greater during the first week of therapy than in subsequent weeks. Further, we have demonstrated in healthy volunteers that 7-day treatment with both citalopram and reboxetine produces positive biases in measures of emotional perception and memory, suggesting that therapeutically relevant changes in neuropsychological function are indeed apparent during the first week of antidepressant administration (38).

Our participants showed no subjective sense of impaired reward to chocolate administration as judged by their ratings of “wanting” or “liking,” even though the corresponding neural response was substantially diminished. This suggests that impaired neural processing of reward does not necessarily become the subject of conscious awareness, although it could still presumably influence behavior. For example, longer-term SSRI treatment is associated with significant weight gain in clinical populations (39) which would not be predicted from the known effects of serotonin on appetite (40). However, using an experimental paradigm similar to our own, Stice *et al.* (41) showed an association between obesity and decreased neural response in striatum to food reward, suggesting that obese subjects might overeat to compensate for the reward deficit. A similar mechanism could explain SSRI-induced weight gain during continued treatment.

Our findings indicate that the undoubtedly clinically useful effects of SSRIs in a wide range of psychiatric conditions characterized by painful and disabling negative affect (42,43) may need to be balanced against their inhibitory effects on the neural responses to reward. As noted earlier, it could be the case that in depressed patients, in whom neural responses to reward appear to be lowered before treatment, SSRIs might in some way produce different effects on reward processing than those seen here in healthy participants. Larger studies in depressed patients and healthy control subjects, employing a within-subject design (pre- and posttreatment) are necessary to test this proposal.

Another relevant point is that although our data agree with plausible neurobiological hypotheses concerning the differential roles of serotonin and catecholamines in antidepressant action (4,10), clinical studies in patients have not revealed consistent differences between serotonin and noradrenergic potentiating antidepressant in their ability to treat specific symptom domains in depressed patients (44). In addition, the important functional interactions between serotonin and catecholamine pathways means that effects of pharmacologically selective agents will not be confined to a single neurotransmitter system (45). Nevertheless, recent pooled analyses suggest that although the catecholaminergic antidepressant bupropion may be slightly less effective than SSRIs in treating depression associated with high levels of anxiety, it appears superior in resolving symptoms of sleepiness and fatigue (46,47). Finally, although SSRI treatment has been associated with emotional blunting in some patients (6,40), it is not established that the incidence of this phenomenon is greater with SSRIs than with other antidepressants acting through contrasting pharmacologic mechanisms.

If it is, in fact, the case that the inhibitory effect of SSRI treatment on neural responses to reward limits the potential effectiveness of SSRIs in the management of depression, it might be possible to improve the efficacy of SSRIs through the recruitment of pharmacologic mechanisms that enhance neural responses to reward as well as diminishing responses to aversive stimuli. A straightforward way to achieve this could be the use of serotonin and noradrenaline reuptake inhibitors (SNRIs) such as duloxetine and venlafaxine. Another strategy would be to add a drug such as bupropion to SSRI therapy. However, although these strategies may produce some benefits over SSRI monotherapy (48,49), overall their effectiveness appears modest (50,51). In this respect, it is not clear that noradrenergic or dopaminergic potentiation has the ability to “reverse” the major inhibitory effect of SSRI treatment on neural reward mechanisms demonstrated by our study. Further investigations examining the effects on reward of SNRIs as well as SSRI combination treatments are needed to address this question.

## Figures and Tables

**Table 1:** Group Demographic and Psychosocial Measures

Measure	Citalopram (*n* = 15)	Reboxetine (*n* = 15)	Placebo (*n* = 15)
Age, years	24.8 (4.8)	25.1 (4.2)	25.2 (5)
Sex (M/F)	7/8	7/8	7/8
BDI	2.5 (3.1)	2.21 (2.8)	1.2 (1.3)
Trait	43 (5.5)	32.3 (7.3)	31.7 (4.8)
FCPS	127 (13)	138.2 (14)	136.6 (14)
SHAPS	22.5 (4.5)	21.9 (4.7)	20.4 (5.5)
BMI	23.7 (3.6)	21.6 (2.2)	24 (2.8)
EAT	4.7 (5)	3 (3)	4.5 (4.2)
Chocolate Craving	6.4 (1.8)	5.9 (2.1)	4.7 (2)
Chocolate Liking	8 (1.5)	8.2 (1.43)	8.3 (1.4)
Chocolate Frequent Eating	1.06 (.3)	.82 (.45)	.78 (.33)

Data are mean (SD) unless otherwise noted. One-way analyses of variance; *p* > .07. BDI, Beck Depression Inventory; BMI, Body Mass Index; EAT, Eating Attitudes Test questionnaire; FCPS, Fawcett Clarke Pleasure Scale; SHAPS, Snaith-Hamilton Pleasure Scale.

**Table 2:** Regions Showing Significant Effect of Treatment on Each Condition Relative to Placebo

Brain Region	MNI Coordinates			*Z* Score	Significance (*p* Value)
	*x*	*y*	*z*		
Chocolate in Mouth: Placebo > Citalopram					
Ventral Striatum	−6	10	−4	4.28	.001
	−8	14	−4	4.22	.001^a^
Sight of Chocolate: Placebo > Citalopram					
Mid OFC	26	32	−10	4.22	.003
Ventral Striatum	6	14	−8	3.79	< .001
VMPFC/MOFC	2	44	−14	3.51	.009
Chocolate in Mouth with Sight of Chocolate: Reboxetine > Placebo					
MOFC/Frontal Pole	10	42	−30	3.01	.05^a^
Strawberry in Mouth: Placebo > Citalopram					
Insula	40	−6	−8	4.41	< .001
Strawberry in Mouth: Placebo > Reboxetine					
LOFC	46	34	−6	4.39	.05
Sight of Moldy Strawberries: Placebo > Citalopram					
LOFC/Anterior Insula	32	26	−4	5.12	.002
Insula	40	−2	−8	4.2	.03
Strawberry in Mouth with Sight of Strawberry: Placebo > Citalopram					
LOFC/Anterior Insula	30	26	2	4.22	< .001

*p* value clusters whole-brain fully corrected (family-wise error). Cing, cingulate; LOFC, lateral orbitofrontal cortex; MNI, Montreal Neurological Institute; MOFC, medial orbitofrontal cortex; VMPFC, ventral medial prefrontal cortex.

a False discovery rate small volume correction.

**Table 3:** Regions Showing Direct Comparison of Treatment Effects

Brain Region	MNI Coordinates			*Z* score	Significance (*p* Value)
	x	y	z		
Chocolate in Mouth: Reboxetine > Citalopram					
Ventral striatum	−6	12	−4	4.64	.07
Putamen	−22	14	−10	4.29	.005
Caudate	10	22	−2	4.85	<.001
MOFC	2	32	−24	3.44	.01^a^
Sight of Chocolate:					
Ventral striatum	8	10	−4	4.02	.001
Insula	38	−6	−6	5.02	<.001
Pregenual cing	0	48	−2	4.66	<.001
Amygdala	18	−8	−16	4.47	.006
Strawberry in Mouth:					
Insula	−38	8	−12	4.52	<.001

*p* value clusters whole brain fully corrected (family-wise error). MNI, Montreal Neurological Institute; MOFC, medial orbitofrontal cortex; cing, cingulate.

a False discovery rate small volume correction.
