# Supplementary material for: Diminished Neural Processing of Aversive and Rewarding Stimuli During Selective Serotonin Reuptake Inhibitor Treatment
Source: Biol Psychiatry. 2010 Mar 1;67(5):439–45. doi: 10.1016/j.biopsych.2009.11.001 (PMC2828549; doi:10.1016/j.biopsych.2009.11.001)
Supplement: Supplementary data [file mmc1.pdf]

Supplemental Information

Table S1. Stimuli

**Condition 1**	chocolate in the mouth + gray visual stimulus	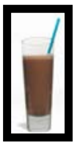 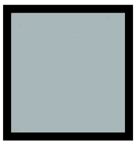
**Condition 2**	picture of chocolate	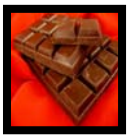
**Condition 3**	chocolate in the mouth + a picture of chocolate	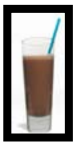 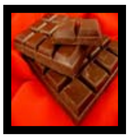
**Condition 4**	strawberry in the mouth + gray visual stimulus	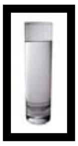 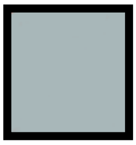
**Condition 5**	picture of moldy strawberries	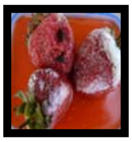
**Condition 6**	strawberry in the mouth + a picture of moldy strawberries	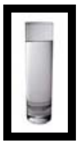 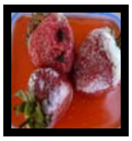
**Rinse condition**	tasteless rinse control solution + gray visual stimulus	

Notes: The term 'chocolate in the mouth' refers to the intra-oral delivery through a teflon tube of 0.5 ml of a fine liquid chocolate, which was identical for all such trials, and which could not be seen by the subject. The term 'strawberry in the mouth' refers to the intra-oral delivery of 0.5 ml of an unpleasant strawberry flavored drink through a tube that also could not be seen by the subject. The term 'picture of chocolate' refers to a picture of a bar of brown (i.e., milk chocolate) shown on the display screen, and the term 'picture of moldy strawberries' to a picture of moldy strawberries.

TABLE S2. Subjective state ratings before and after 7 days of treatment with citalopram, reboxetine or placebo. There were no significant effects of treatment group on any of the measures.

**Measure**	**Citalopram (*n*=15)**		**Reboxetine (*n*=15)**		**Placebo (*n*=15)**	
	**Mean (SD)**		**Mean (SD)**		**Mean (SD)**	
	Pre-treat	Post-treat	Pre-treat	Post-treat	Pre-treat	Post-treat
**Alertness**	65.8 (12.2)	54.6 (26)	71.6 (12.5)	58.6 (22)	63.8 (18)	57.5 (28)
**Disgust**	12.3 (14.4)	9.8 (16.9)	8.1(6.5)	14.3 (27.5)	5.58 (6.4)	6.1 (7.2)
**Drowsiness**	29.6 (23)	9 (6.4)	27.3 (21.4)	27 (34)	27.4(20)	16.8 (15.5)
**Anxiety**	11.8 (7.2)	15.5 (16)	13.7 (12.6)	16.3 (24.6)	14.2 (22)	7.8 (8.8)
**Happiness**	7.8 (7.3)	12.7 (9.5)	4.9 (9.3)	15.6 (28.6)	3.6 (2.9)	4.5 (5.4)
**Nausea**	71 (13.7)	60 (19)	76.3 (14)	66.8 (14)	77.4 (13)	72.8 (12.6)
**Sadness**	12.5 (14.3)	9.9 (13.2)	8.4 (9.24)	12.5 (18.8)	7 (5.8)	6.8 (6)
**State Anxiety**	32.5 (6.7)	36.3 (10.2)	32.4 (10.6)	34.6 (13.5)	31 (8.5)	27.7 (6.6)

SD, standard deviation; Repeated measures ANOVAs all $p > 0.1$

TABLE S3. Regions showing main effect of task irrespective of treatment for all subjects.

Brain Region	Montreal Neurological Institute (MNI) Coordinates			Z-score	Significance (*p*-value)
	X	Y	Z		
**Chocolate in the mouth**					
Ventral striatum	-10	14	-4	6.33	<0.001
Anterior insula	-34	16	0	6.63	<0.001
Mid OFC	22	36	-14	4.67	0.007
Pregenual cingulate	-4	20	44	5.88	<0.001
Frontal pole	-38	46	12	5.57	<0.001
**Sight of chocolate**					
Caudate	-10	12	0	6.01	<0.001
Anterior insula	-32	12	2	7.1	<0.001
	34	24	2	6.24	<0.001
Middle OFC	26	34	-12	5.68	<0.001
Frontal pole	-36	50	24	6.16	<0.001
**Strawberry in the mouth**					
Lateral OFC/Insula	34	24	-4	7.4	<0.001
Caudate	10	14	0	6.06	<0.001
Ventral striatum	10	12	-6	6.06	<0.001
Pregenual cingulate	4	20	42	7.11	<0.001
**Sight of moldy strawberries**					
Insula	-32	18	6	6.93	<0.001
	40	20	-2	6.72	<0.001
Superior frontal gyrus	2	20	60	5.75	<0.001

OFC, orbitofrontal cortex; *p*-values clusters whole brain fully corrected (FWE)

Table S4. Subjective ratings of pleasantness, intensity and wanting for each of the 6 conditions taken during the scanning session for citalopram, reboxetine and placebo groups

PLEASANTNESS

	Citalopram	Reboxetine	Placebo		Citalopram	Reboxetine	Placebo
**CHOC TASTE**	1.04	1.10	1.14	**STRAW TASTE**	-0.93	-0.68	-0.94
STDEV	0.54	0.41	0.32	STDEV	0.81	-0.68	0.60
SEM	0.14	0.10	0.08	SEM	0.21	0.26	0.15
**CHOC PIC**	0.97	1.07	1.13	**STRAW PIC**	-1.16	-1.13	-1.06
STDEV	0.58	0.57	0.32	STDEV	0.48	-1.13	0.42
SEM	0.15	0.15	0.08	SEM	0.12	0.14	0.11
**CHOC BOTH**	1.23	1.35	1.28	**STRAW BOTH**	-1.21	-1.19	-1.37
STDEV	0.51	0.50	0.37	STDEV	0.62	-1.19	0.33
SEM	0.13	0.13	0.10	SEM	0.16	0.17	0.09

INTENSITY

	Citalopram	Reboxetine	Placebo		Citalopram	Reboxetine	Placebo
**CHOC TASTE**	1.77	1.80	2.00	**STRAW TASTE**	2.07	1.99	2.05
STDEV	0.66	0.71	0.73	STDEV	0.72	0.71	0.74
SEM	0.17	0.18	0.19	SEM	0.19	0.18	0.19
**CHOC PIC**	1.57	0.61	1.29	**STRAW PIC**	1.71	1.78	1.43
STDEV	0.72	0.81	0.61	STDEV	0.86	0.92	0.71
SEM	0.19	0.21	0.16	SEM	0.22	0.24	0.18
**CHOC BOTH**	2.15	2.25	2.24	**STRAW BOTH**	2.31	2.38	2.57
STDEV	0.75	0.89	0.78	STDEV	0.79	0.91	0.69
SEM	0.19	0.23	0.20	SEM	0.20	0.24	0.18

WANTING

	Citalopram	Reboxetine	Placebo		Citalopram	Reboxetine	Placebo
**CHOC TASTE**	0.98	0.96	1.01	**STRAW TASTE**	-1.05	-0.87	-1.11
STDEV	0.55	0.52	0.43	STDEV	0.82	0.93	0.50
SEM	0.14	0.13	0.11	SEM	0.21	0.24	0.13
**CHOC PIC**	0.98	0.94	1.08	**STRAW PIC**	-1.24	-1.23	-1.15
STDEV	0.58	0.69	0.41	STDEV	0.45	0.59	0.47
SEM	0.15	0.18	0.10	SEM	0.12	0.15	0.12
**CHOC BOTH**	1.14	1.22	1.13	**STRAW BOTH**	-1.29	-1.32	-1.44
STDEV	0.56	0.61	0.52	STDEV	0.48	0.57	0.31
SEM	0.14	0.16	0.13	SEM	0.13	0.15	0.08

Choc, chocolate; straw, strawberry; STDEV, standard deviation; SEM, standard error of the mean
